# Bidirectional best hit *r*-window gene clusters

**DOI:** 10.1186/1471-2105-11-S1-S63

**Published:** 2010-01-18

**Authors:** Melvin Zhang, Hon Wai Leong

**Affiliations:** 1 School of Computing, National University of Singapore, Computing 1, 13 Computing Drive, Singapore 117417, Republic of Singapore

## Abstract

**Background:**

*Conserved gene clusters *are groups of genes that are located close to one another in the genomes of several species. They tend to code for proteins that have a functional interaction. The identification of conserved gene clusters is an important step towards understanding genome evolution and predicting gene function.

**Results:**

In this paper, we propose a novel pairwise gene cluster model that combines the notion of bidirectional best hits with the *r*-window model introduced in 2003 by Durand and Sankoff. The bidirectional best hit (BBH) constraint removes the need to specify the minimum number of shared genes in the *r*-window model and improves the relevance of the results. We design a subquadratic time algorithm to compute the set of BBH *r*-window gene clusters efficiently.

**Conclusion:**

We apply our cluster model to the comparative analysis of *E. coli *K-12 and *B. subtilis *and perform an extensive comparison between our new model and the gene teams model developed by Bergeron *et al*. As compared to the gene teams model, our new cluster model has a slightly lower recall but a higher precision at all levels of recall when the results were ranked using statistical tests. An analysis of the most significant BBH *r*-window gene cluster show that they correspond to known operons.

## Background

It is well-known that the differences between the genomes of extant species can be attributed to both small and large-scale mutations [1]. Large-scale mutations or rearrangements are relatively rare but they affect the content and order of the genomes, thereby obscuring the relationship between species. Comparison of multiple genomes based on their gene orders -- the sequence of genetic markers -- reveal segments with homologous gene content. These segments are commonly referred to as *conserved gene cluster*.

These homologous regions may have resulted from functional pressure to keep sets of genes in close proximity across multiple species. The most well studied examples are co-transcribed genes, also known as operons, in prokaryotes [2]. In addition, [3] showed that genes in the same cluster tend to code for proteins that have a functional interaction. Gene clusters can also result from the evolutionary proximity of the genomes being analyzed. Such clusters provide important phylogenetic information which can be used to infer the gene order of the ancestral genomes [4] and identify ancestral homologs among genes from the same family [5].

Intuitively, a conserved gene cluster represents a compact region which contains a large proportion of homologous genes separated by regions that do not contain any shared homologs. Developing a formal definition of such clusters is a non-trivial task due to conflicting cluster properties. Two cluster definitions that are commonly used in practice are max-gap clusters and *r*-window clusters [6].

The gene teams model [7,8], a formalization of max-gap clusters, allows for gaps of length at most *δ *between adjacent genes in a team. [9] further generalized the model by introducing a quorum parameter, *q*, to allow gene teams which may be found in at least *q *input genomes. Efficient algorithms to find all gene teams have been proposed in [7,8,10].

While the gene teams model imposes a constraint on the distance between adjacent genes in a cluster, the size (number of genes) and length (distance between the two furthest genes) of the resulting clusters are unbounded. In contrast, under the *r*-window model [11], clusters have length at most *r *and contains at least *k *genes. The statistical properties of *r*-window gene clusters are also better understood and the significance of discovered clusters can be evaluated using statistical tests proposed in [11]. Exact computation of the significance of gene teams is still an open problem, but upper and lower bounds have been developed in [12].

The *r*-window model was first used in the study of block duplications [13,14], by comparing all pairs of windows of length *r*. To the best of our knowledge, no formal algorithms has been presented for computing all *r*-window gene clusters. In addition, although several models of conserved gene clusters have been proposed in the literature, there are few published results which compares different models empirically on real genomes. [6] laid the groundwork by providing a characterization of the desirable properties of gene clusters and a detailed analysis of the difference between max-gap clusters based on the gene teams model and those produced by heuristics.

In this paper, we propose an improvement to the *r*-window gene cluster model [11] by imposing the bidirectional best hit (BBH) criteria from sequence homology. We formulate the clustering problem formally and design an efficient subquadratic time algorithm to compute all BBH *r*-window gene clusters between two gene orders based on a sliding window technique. Finally, we give an empirical comparison between our new cluster model and the gene teams model.

### Notation

Our model of a genome is as a sequence of genomic markers for which homology information across the genomes of interest are available. The most common and well annotated type of genomic markers are protein coding genes. A homology family is a groups of genes that are descended from a common ancestral gene [15].

Let Σ denote the set of homology families. We are interested in the homology families that contains at least one gene in each of the genomes under consideration. A *gene*, *g*, is denote by *f*^*p *^where *f *∈ Σ is the homology family which the gene belongs to and *p *∈ ℝ is the position the gene.

The *distance *between two genes *g *= *a*^*p *^and *h *= *b*^*q*^, Δ(*g*, *h*), is simply the absolute difference in their position, *i.e*. |*p-q*|. This represents the number of elements located between the two genes of interest. In our experiments, gene positions are assigned based on the index of the gene in the complete genome. Hence, the distance between two genes reflect the number of genes between them. We make use of the notion of distance to constrain the maximum length of a gene cluster.

A *gene order*, *G*, is a sequence of genes ⟨*g*_1_, *g*_2_, ..., *g*_*n*_⟩ in increasing order of their position. A uni-chromosomal genome can be directly represented as a gene order. Genomes with multiple chromosomes can be represented as a gene order by concatenating the chromosomes together in an arbitrary order and inserting an appropriate gap to separate genes from different chromosomes.

A *r-window *[11], *G *[*i*, *j*] = ⟨*g*_*i*_, *g*_*i*+1_, ..., *g*_*j*_⟩, on a gene order *G *is a substring of *G*. The length of a window, defined by the distance between the first and last gene, is at most *r*, *i.e*. Δ(*g*_*i*_, *g*_*j*_) ≤ *r*. In [11], a gene cluster is defined as a set of *k *genes that are found in a *r*-window. When extended to two gene order, this definition imposes a constraint on the number of common genes between two *r*-windows. However, it is unclear how to determine the minimum number of genes in a gene cluster as it is affected by the actual length of the clusters and the evolutionary distance between the genomes. Too low a value will introduce too many false positives, while a more conservative value may exclude weakly similar clusters.

### Problem definition

In this paper, we adopt a different constraint on the *r*-window gene cluster, namely, bidirectional best hit. This circumvents the problem of having to decide the number of common genes in a cluster by making use of the relative similarities between the *r*-windows. The bidirectional best hit (BBH) criteria is routinely used when identifying homologous DNA sequences between two species using BLAST. We feel that it is natural to extend this criteria to the identification of conserved gene clusters, as they are essentially homologous chromosomal segments.

In order to apply the BBH criteria, we will need a measure of similarity between two windows. A straightforward method is to count the number of genes that come from a common homology family, we call this the shared gene count.

**Definition 1 **(Shared gene count). *Given two windows, w*_*G *_= *G *[*i*, *j*] *and w*_*H *_= *H*[*k*, *l*], *the *shared gene count *of w*_*H *_*with respect to w*_*G *_*is the number of genes in w*_*H*_* that comes from a homology family that is also present in w*_*G*_.

Based on the shared gene count, we define the notion of a *best hit *from one genome to another genome.

**Definition 2 **(Best hit). *Given a window w*_*G *_*and a collection of windows W*_*H*_, *the *best hit *is a window w*_*H *_*in W*_*H *_*with the highest shared gene count with respect to w*_*G*_. *If there are multiple windows with the same shared gene count, then the best hit is the shortest one*.

Although we define the best hit in terms of the shared gene count, it is possible to replace it with other more sophisticated similarity measures. The simplicity of the shared gene count makes it easy to understand and allows us to design an efficient algorithm to find all clusters.

The following definition formally defines our *BBH r-window gene cluster *model.

**Definition 3 **(BBH *r*-window gene cluster). *Given two gene orders, G and H, and a maximum window length, r, let W*_*G *_*denote the set of r-windows in G and W*_*H *_*the set of r-windows in H. A pair of r-windows*, (*G *[*i*, *j*], *H*[*k*, *l*]) ∈ *W*_*G *_× *W*_*H*_, *is a *bidirectional best hit *r*-window gene cluster *if it satisfies the following properties:*

• *H *[*k*, *l*] *is the best hit for G *[*i*, *j*]

• *G *[*i*, *j*] *is the best hit for H *[*k*, *l*]

• (*G *[*i*, *j*], *H *[*k*, *l*]) *is maximal with respect to substring inclusion, i.e. there is no other BBH r-window gene cluster *(*G *[*i'*, *j'*], *G *[*k'*, *l'*]) *such that i' ≤ i *≤ *j *≤ *j' *and *k' *≤ *k *≤ *l *≤ *l'*.

A BBH *r*-window gene cluster is *trivial *if it contains a single pair of genes.

**Example**. *Consider the following two gene orders*,

*where the letters represent homology families and the superscripts denote the position*.

*The non-trivial BBH *3-*window gene clusters of G and H are:*

• (⟨*c*^5^, *d*^6^⟩, ⟨*c*^1^, *a*^3^, *d*^4^⟩)

• (⟨*e*^9^, *c*^10^, *b*^11^⟩, ⟨*b*^6^, *e*^7^, *b*^8^, *c*^9^⟩)

Given the above model of conserved gene clusters, the task is to compute all occurrences of BBH *r*-window gene clusters between two given gene orders. Formally, we define the BBH *r*-window gene clustering problem as follows:

**BBH ***r***-window gene clustering problem **Given two gene order, *G *= ⟨ *g*_1_, *g*_2_, ..., *g*_*n*_⟩, *H *= ⟨*h*_1_, *h*_2_, ..., *h*_*m*_⟩, and a maximum window length, *r*, compute the set of non-trivial BBH *r*-window gene clusters.

## Methods

In this section, we describe our algorithm for solving the BBH *r*-window clustering problem described in the previous section. We start off with a simple quadratic time algorithm and show how it can be improved using a sliding window technique. Finally, we present an efficient data structure that allows us to obtain a subquadratic time algorithm.

### A simple quadratic time algorithm

A straightforward algorithm is to generate both sets of windows *W*_*G *_and *W*_*H*_, then for each window in *W*_*G*_, compute its best hit in *W*_*H *_by going through each window in *W*_*H *_and vice versa. For simplicity, we assume that there are at most *r *genes in a window of length *r*. Therefore, the size of *W*_*G *_and *W*_*H *_is *O*(*nr*) and *O*(*mr*) in the worst case and comparing two windows take *O*(*r*) time. This simple algorithm has a time complexity of *O*(*nmr*^3^).

### A sliding window algorithm

We first show how we can find the best hits for each window in *W*_*G *_efficiently. Finding the best hits for windows in *W*_*H *_is done in the same way. After that, we go through the hits and keep only the bidirectional best hits.

One problem in the previous algorithm is that many of the comparisons between two windows would result in a shared gene count of zero. Therefore, instead of storing all the *r*-windows, we generate them one-the-fly to avoid comparing two windows with no common homology family.

We enumerate the windows in *W*_*G *_by starting from each gene and incrementally add genes in increasing order of their position as long as the window length is less than or equal to *r*. We use a data structure *T *to maintain the set of windows *W*_*H *_that have a non zero shared gene count with respect to the current window in *W*_*G*_.

Each time we consider a different window *w*_*G*_, we need to update our data structure by adding the corresponding genes in *H *from the same family to our data structure. To determine the list of genes to be added, we preprocess *H *to compute the list of genes for each homology family. Finally, for each window *W*_*G*_, we make use of our data structure to determine the best hit in *H*.

The pseudo code for this algorithm is shown in Algorithm 1.

Putting it all together, we first find the best hits from *G *to *H *and vice versa, then filter the results to only retain the bidirectional best hits. We store the best hits from *H *to *G *in a hash table and for each best hit from *G *to *H*, we access the table to check if it is also a best hit from *H *to *G*.

The pseudocode for the algorithm is shown in Algorithm 2.

#### Data structure for W_*H*_

Observe that for a given window *W*_*G *_in *W*_*G*_, most of the windows in *W*_*H *_do not have any genes in common with *W*_*G*_. Hence, instead of finding the best hit by checking against all windows in *W*_*H*_, we only represent

**Algorithm 1 **BestHitWindows(*G*, *H*, *r*)

**Ensure: **Determine for each window in *W*_*G *_the best hit in *W*_*H*_

   *BH *:= ∅ {set of best hits from *G *to *H*}

   {Determine list of genes for each family in *H *and store in *gs*}

   **for ***i *from 1 to *n*_*H *_**do**

      *h*_*i *_:= *i*th gene in *H*

      *f*_*i *_:= family of *h*_*i*_

      *gs *[*f*_*i*_] := *gs *[*f*_*i*_] ∪ {*h*_*i*_}

   **end for**

   {Enumerate *r*-windows in G and compute best hits}

   **for ***i *from 1 to *n*_*G *_**do**

      {*g*_*i *_is the *i*th gene in *G*}

      *e *:= *i *- 1

      *w*_*g *_:= ∅

      initialize *T*

      **while Δ **(*g*_*i*_, *g*_*e*+1_) ≤ *r ***do**

         *e *:= *e *+ 1

         *w*_*g *_= *w*_*g *_∪ {*g*_*e*_}

         *f*_*e *_:= family of *g*_*e*_

         **for **each gene *g *∈ *gs *[*f*_*e*_] **do**

            insert(*T*, *g*)

         **end for**

         *w*_*h *_:= besthit(*T*)

         *BH *:= *BH *∪ {(*W*_*G*_, *W*_*H*_)}

      **end while**

   **end for**

   **return ***BH*

**Algorithm 2 **BBHWindows(*G*, *H*, *r*)

**Ensure: **Compute the set of BBH *r*-window gene clusters between *G *and *H*

   *BBH *:= ∅ {set of bidirectional best hits}

   *BH *_*G*, *H *_:= BestHitWindows(*G*, *H*, *r*)

   *BH *_*H*, *G *_:= BestHitWindows(*H*, *G*, *r*)

   {Store the best hits from *H *to *G *in a hash table *M*}

   **for **each (*W*_*H*_, *W*_*G*_) in *BH *_*H*, *G *_**do**

      *M *[*w*_*h*_] := *W*_*G*_

   **end for**

   {Compute the bidirectional best hits}

   **for **each (*W*_*G*_, *W*_*H*_) in *BH *_*G*, *H *_**do**

      **if ***M *[*W*_*H*_] = *W*_*G*_**then**

         *BBH *:= *BBH *∪ {(*W*_*G*_, *W*_*H*_)}

      **end if**

   **end for**

   **return ***BBH*

the windows that have at least one gene in common with *W*_*G*_.

This is achieved by storing the genes in *H *that share a family with *w*_*g *_in a balanced binary search tree, *T*, using the position of the gene as the key. Each gene represents the start of a window, thus each node in the data structure represents a window of length *r *in *H*.

We need to be able to insert/delete genes in this structure and find the largest window. To find the largest window efficiently, we maintain the shared gene count, *s*, of each window as an additional attribute of each node.

Insertion and deletion of a gene follows from the algorithm for standard binary search tree. Unfortunately these two operations cause the shared gene count of up to *r *contiguous windows to change. Instead of updating these windows one by one, we make an update to the roots of the subtrees that contains only these windows to indicate the change in shared gene count to all the windows in the subtrees. For this to work, we need to keep track of the range of windows in a subtree by storing the minimum and maximum position of genes, (min_*p*_, max_*p*_), and the adjustment to the shared gene count, Δ_*s*_. This is similar to the canonical decomposition technique used in segment trees [16]. Hence, the number of nodes affected is at most *O*(lg |*T|*), where |*T*| is the number of nodes in the tree.

To find the window with the highest shared gene count, we need to keep store the maximum shared gene count in each subtree. Then the maximum shared gene count in the whole tree is found in the root. Finding the best hit is done by traversing only those nodes whose maximum shared gene count is equal to the maximum in the whole tree. The complexity of this step is therefore *O*(lg |*T*|).

In summary, to make the three operations efficient, we augment each node of the tree with the following attributes:

*s *-- shared gene count for the window of length *r *starting at this gene

max_*s *_-- maximum shared gene count of windows in this subtree

(min_*p*_, max_*p*_) -- minimum and maximum position of genes in this subtree; used to determine the windows under this subtree

Δ_*s *_-- adjustment in shared gene count made to all windows in this subtree

When rotations are necessary to maintain the balance of the tree, the additional attributes in the nodes can be updated in constant time as they can be computed from the attributes in the left and right subtrees.

#### Time complexity

The first part of the algorithm determine the list of genes in *H *for each homology family. This has a worst case time complexity of *O*(*m*). The complexity of the operations on the data structure *T*, depends on its size, which is *O*(*r*). Hence, the complexity of determining the best hits for each window in *W*_*G *_is *O*(*m *+ *nr *lg *r*) and the complexity for determining the best hits in both directions is *O*((*n *+ *m*)*r *lg *r*). The number of results for BestHitWindows(*G*, *H*, *r*) and BestHitWindows(*H*, *G*, *r*) is *O*(*nr*) and *O*(*mr*) respectively. Creating the associative array to index the best hits from *H *to *G *takes *O*(*mr*) time on average using a hash table. Going through the best hits from *G *to *H *and keeping only the bidirectional best hits takes *O*(*nr*) time on average, assuming expected *O*(1) time to access the hash table.

Therefore, the time complexity of the whole algorithm is dominated by the time taken to compute the best hits which is *O*((*n *+ *m*)*r *lg *r*).

## Results and discussion

We investigated the power of our BBH *r*-window model by applying it to the analysis of conserved gene clusters between *E. coli *K-12 and *B. subtilis *and comparing our results with that obtained by [8] based on the gene teams model [7].

It has been shown that in prokaryotic genomes, conserved gene clusters can be used to identify co-transcribed genes, known as operons [8]. However, we note that methods for finding operons often make use of machine learning techniques that incorporate multiple sources of information in addition to the spatial proximity of the genes. Our experiments indicate that the kind of spatial conservation modelled by conserved gene clusters provides some signal that can be used for identifying operons.

We implemented our subquadratic time algorithm, which finds all BBH *r*-window gene clusters between two gene orders, in Java. All computations were performed on a Intel Core 2 Duo E6550 (2.33 GHz) processor with 2 GB of RAM running Linux.

In the *E. coli *K-12 and *B. subtilis *dataset from [8], homology families were assigned to each gene based on the COG database [17]. Originally, there are 4289 genes in *E. coli *K-12 and 4100 genes in *B. subtilis*, after removing the genes which are unique to each genome, we are left with 2339 genes in *E. coli *K-12 and 2332 genes in *B. subtilis *from 1137 homology families. Gene positions are assigned based on the index of the gene in the complete genome, thus the distance between two genes represents the number of intervening genes, including those genes that are not shared between the two genomes.

We computed the BBH *r*-window gene clusters between these two genomes and compared our results against known *E. coli *K-12 operons from RegulonDB [18]. As it is difficult to obtain an exact match, we compute the Jaccard score for each operon based on the Jaccard coefficient between the operon and one of the clusters we computed.

**Definition 4 **(Jaccard coefficient [19]). *The *Jaccard coefficient *of two sets o and c is defined as *. *It gives value between zero and one. A value of one indicates a perfect match*.

**Definition 5 **(Jaccard score). *The *Jaccard score *of an operon o with respect to a set of gene clusters C is the highest Jaccard coefficient between o and some cluster in C*.

**Figure 1:**
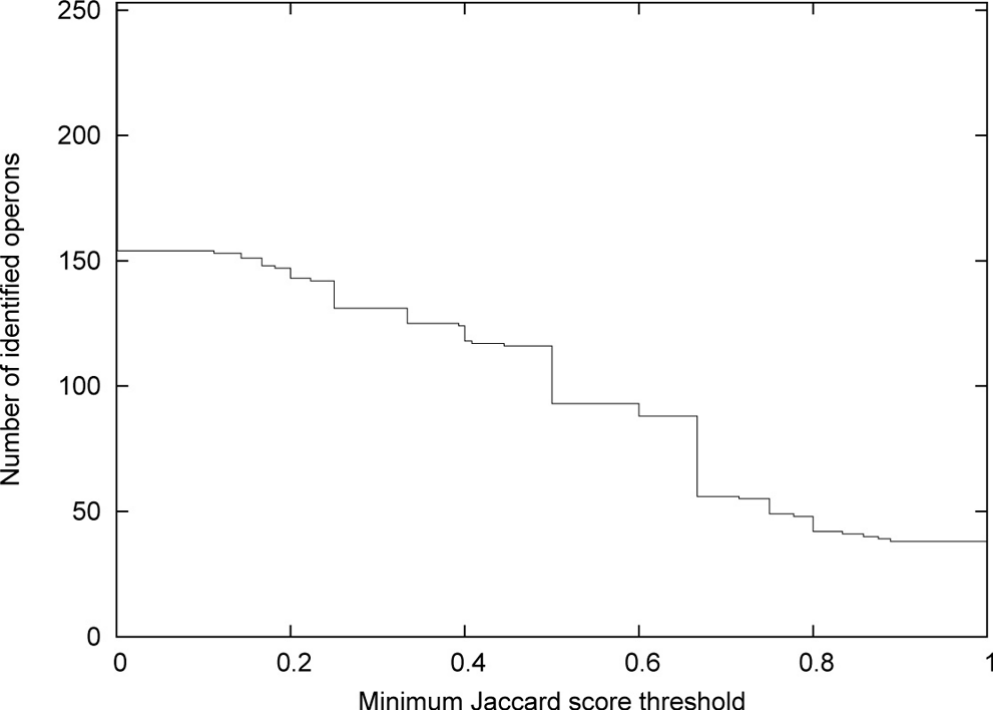
**Effect of Jaccard score threshold**. Plot of the number of identified operons versus Jaccard score threshold for BBH *r*-window gene clusters, where the maximum window length is 6.

We consider an operon to be identified if its Jaccard score is above a certain minimum Jaccard score threshold. Figure 1 shows the number of identified operons for difierent values of the threshold when the maximum window length is 6.

Based on Figure 1, we chose a value of 2/3 for the threshold. There are 253 operons with at least two genes and at least 2/3 of its genes are common to both *E. coli *K-12 and *B. subtilis*. This is an upper bound on the number of operons that can be identified based on the input.

### Effect of window length

Our gene cluster model has a single parameter *r*, which is the maximum length of a window. A natural question that arises is the effect of this parameter on the resulting clusters. We ran our algorithm for a range of window lengths from 1 to 30, each run took approximately 8 seconds to complete.

**Figure 2:**
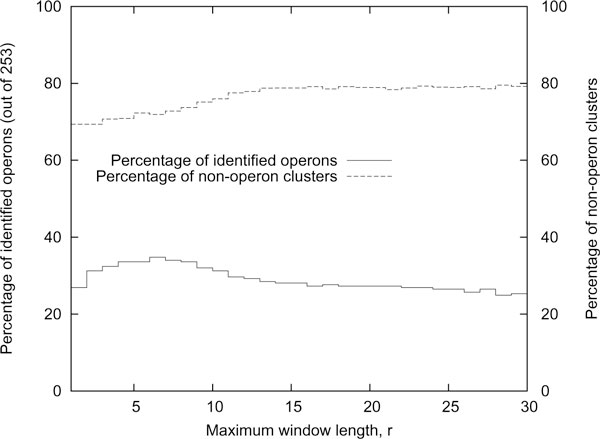
**Effect of window length**. Plot of the percentage of identified operons and percentage of non-operon clusters versus maximum window length for our BBH *r*-window gene clusters model.

As shown in Figure 2, the percentage of identified operons increases as the window length increases from 1 to 6 and decreases for larger values of *r*. At the peak, when the maximum window length is 6, our method identified 34% of the operons (85 out of 253). It is interesting to note that there is a core of about 60 operons that are identified across the entire range of the parameter *r*. The dashed line shows the number of BBH *r*-window gene clusters that are not matched to any operon; it ranges between 70% to 80%. This illustrates the difficulty of using only spatial information to distinguish between the two kinds of genes clusters: those that are due to the evolutionary proximity of the two species and those that are under selective pressure.

### Comparison with gene teams

In this section, we present the first empirical comparison between two different conserved gene cluster models that takes into account gene position and the distance between genes.

**Figure 3:**
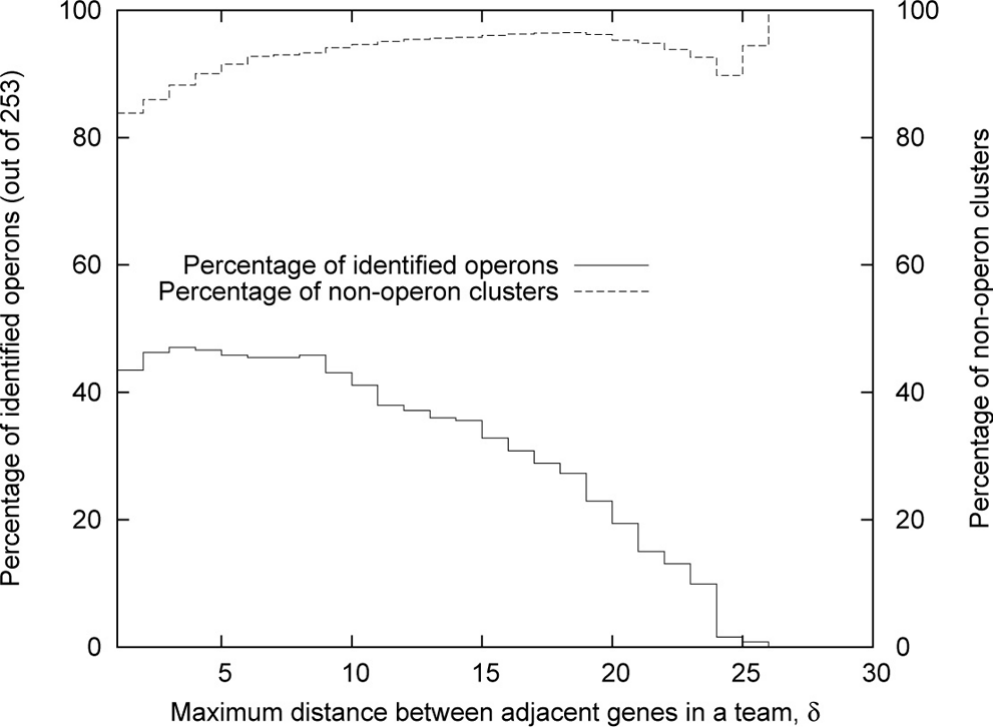
**Effect of maximum gap length**. Plot of the percentage of identified operons and percentage of non-operon clusters versus maximum distance between adjacent genes in a team for the gene teams model.

We compared our results against the gene teams model. The gene teams model has a single parameter *δ*, which is the maximum distance between adjacent genes in a cluster. We computed the gene team tree [20] for our dataset (ignoring singleton teams) and found that a maximum of 47% of the operons (119 out of 253) was identified when *δ *is 3 (see Figure 3).

This is slightly higher than the 34% achieved by our BBH *r*-window model, however, at all parameter values the percentage of non-operon teams is much higher for the gene teams model. This suggests that only a small percentage of the gene teams are identified as operons, due to the property that gene teams always form a partition of the set of genes. In addition, we observe that over the same range of parameter values, variation in the number of identified operons for our BBH *r*-window gene clusters is lower than that for gene teams. This means that our model is more robust to changes to the value of its parameter as compared to the gene teams model.

**Figure 4:**
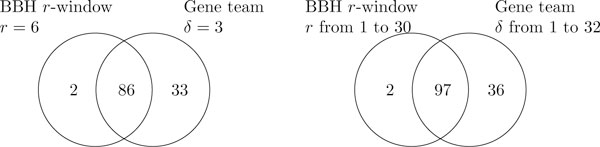
**Overlap in operons identified by the two cluster models**. Venn diagram showing the overlap between the operons identified based our BBH *r*-window gene cluster model and the gene teams model for a single parameter value (*r *= 6, *δ *= 3) and over a range of parameter values (*r *∈ [1, 30], *δ *∈ [1, 32]).

Figure 4 consists of two Venn diagrams which illustrates the overlap between the operons identified by our BBH *r*-window gene cluster model and the gene teams model for a single value of the parameter and over a range of parameter values. Considering a range of different parameter values for both models did not significantly increase the number of identified operons as most operons can be modelled by a small range of parameters.

The operons identified using BBH *r*-window gene cluster are mostly a subset of the ones identified using the gene teams model. Both models agree on a common set of 86 operons. This result is expected since our BBH *r*-window model is more restrictive. Hence, the recall, which is percentage of identified operons, is lower as compared to gene teams.

**Figure 5:**
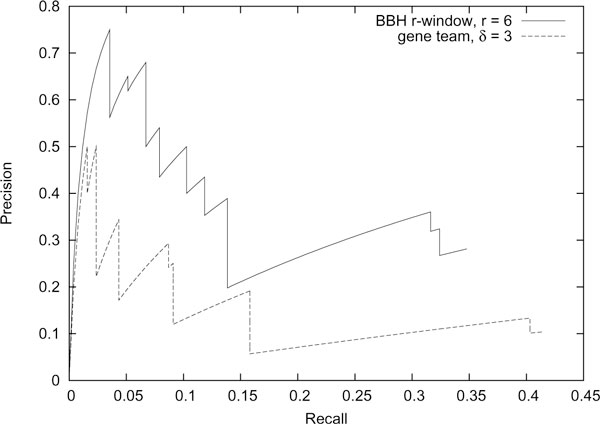
**Comparison of precision versus recall curves**. Precision versus recall curve for BBH *r*-window gene clusters (*r *= 6) and gene teams (*δ *= 3) for identification of *E. coli *K-12 operons.

However, an advantage of *r*-window gene clusters is the availability of exact statistical tests to evaluate the significance of putative clusters. We computed the expected number of *r*-window gene clusters with *k *genes between two random genomes (equation 55 in [11]) and use it to rank the BBH *r*-window gene clusters when *r *is 6. We also ranked each of the gene teams when *δ *is 3 following [8] by using the probability of forming a gene team of size *k *(equation 3 in [8]). Given these two list of ranked gene clusters, we computed the precision and recall at all possible cut-offs. For a set of top *p *gene clusters, *C*_*p*_, and a set of operons, *O*, the precision is defined as |*C*_*p *_∩ *O*|/|*C*_*p*_| and the recall is defined as |*C*_*p *_∩ *O*|/|*O*|. Although our BBH *r*-window gene clusters had a slightly lower recall as compared to gene teams, our model has a much higher precision. Figure 5 plots the precision versus recall curve for both gene cluster models; it clearly shows that at any given recall, the precision of our model is *always *higher than the gene teams model. For example, at a recall of 0.05, 65% of the BBH *r*-window gene clusters match 5% of the identifiable operons, whereas only 20% of the gene teams match the same number operons. Similarly, at a recall of 0.1, 50% of the BBH *r*-window gene clusters match 10% of the identifiable operons, while only 13% of the gene teams match the same number operons.

### Analysis of significant clusters

Table 1 shows nine of the top twelve BBH *r*-window gene cluster in increasing order of log *E*, the logarithm of the expected number of clusters between two random genomes.

**Table 1:** Significant BBH *r*-window gene clusters and corresponding operons. Nine out of the top twelve based on log *E *value and corresponding operons. Numbers in brackets indicate number of genes in the cluster over number of genes in the operon.

log E	BBH *r*-window gene cluster	Operon
-13	atpC, atpD, atpG, atpA, atpH, atpF, atpE, atpB	atpIBEFHAGDC (8/8)

-12	secE, nusG, rplK, rplA, rplJ, rplL, rpoB, rpoC	secE-nusG (2/2), rplKAJL-rpoBC (6/6)

-11	hisG, hisD, hisB, hisH, hisA, hisF, hisI	hisLGDCBHAFI (7/8)

-10	fliE, fliF, fliG, fliH, fliI, fliJ, fliK	fliFGHIJK (7/6)

-9	menE, menC, menB, yfbB, menD, menF	menFD-yfbB-menBCE (6/6)

-9	rbsD, rbsA, rbsC, rbsB, rbsK, rbsR	rbsDACBKR (6/6)

-8	pnp, rpsO, truB, rbfA, infB, nusA, yhbC	metY-yhbC-nusA-infB-rbfA-truB-rpsO-pnp (7/7)

-8	dppF, dppD, dppC, dppB, dppA, yhjX	dppABCDF (6/5)

-7	oppA, oppB, oppC, oppD, oppF	oppABCDF (5/5)

Most of the clusters are an exact match to a specific operon, except for the second cluster which consists of a combination of two operons. Two of our clusters contains an additional gene that is not part of the operon.

The fliE-K cluster includes the additional fliE gene that is not part of the fliF operon. The fliE gene is known to be a monocistronic transcriptional unit that is adjacent to the fliF operon, it forms part of the flagellar of *E. coli *K-12 together with the fliF operon [21]. This evidence supports our grouping of fliE together with the fliF operon.

The cluster matched to the dpp operon contains an addition yhjX gene. yhjX is a hypothetical protein with an unknown function predicted to be a transporter [22]. This prediction gives yhjX a similar function as the dpp operon, which function as a dipeptide transporter, and gives support to our cluster.

## Conclusion

In this paper, we proposed a novel variant of the *r*-window gene cluster model based on the bidirectional best hit constraint. The bidirectional best hit criteria is most commonly used for identifying families of homologous DNA sequences from BLAST hits. We extend this notion to identify homologous chromosomal segments/conserved gene clusters.

We developed a simple quadratic time algorithm to compute the set of BBH *r*-window gene clusters from two genomes and show how it can be improved to a subquadratic time algorithm. The key insight is to use a segment tree like data structure for maintaining a set of windows and reporting the best hit.

Our comparative analysis of the *E. coli *K-12 and *B. subtilis *dataset showed that the operons identified by our more restrictive BBH *r*-window model is a subset of the operons identified by the gene teams model. However, as a result of the BBH constraint, we were able to achieve a higher level of precision at all levels of recall as compared to the gene teams model. In addition, a detailed analysis of the most significant BBH *r*-window gene cluster show that the top ranking results match well to known *E. coli *K-12 operons.

## Competing interests

The authors declare that they have no competing interests.

## Authors' contributions

MZ came up with the BBH *r*-window gene cluster model, designed the algorithms, and drafted the manuscript. HWL contributed to research discussions and made improvements to the analysis and presentation of the results. All authors read and approved the final manuscript.
